# ‘Like jumping off a ledge into the water’: A qualitative study of trauma‐focussed imaginal exposure for hearing voices

**DOI:** 10.1111/papt.12372

**Published:** 2021-11-19

**Authors:** Natalie Feary, Rachel Brand, Anne Williams, Neil Thomas

**Affiliations:** ^1^ Centre for Mental Health Swinburne University of Technology Melbourne Victoria Australia; ^2^ Department of Nursing and Allied Health Swinburne University of Technology Melbourne Victoria Australia; ^3^ Alfred Hospital Melbourne Victoria Australia

**Keywords:** auditory verbal hallucinations, cognitive behavioural therapy for psychosis, hearing voices, prolonged exposure, schizophrenia, trauma‐focussed interventions

## Abstract

**Objective:**

There is growing evidence of a link between the experience of hearing voices and past traumatic events, and trauma‐focussed psychological interventions are being applied to hearing voices as an emerging treatment direction. To inform the ongoing development and implementation of this application, there is a need to understand clients’ therapy experiences.

**Design:**

Qualitative study exploring the experience of people who received an intervention for voices linked to a previous traumatic event.

**Method:**

Ten participants experiencing voices with some connection to a previous traumatic event participated in individual semi‐structured interviews following six sessions of imaginal exposure, an exposure‐based trauma‐focussed intervention. Participant responses were analysed using thematic analysis.

**Results:**

Participants reported a range of benefits from the intervention, including improved mental health, reduction of distressing voice‐hearing experiences, and increased clarity of the traumatic event. The therapy was perceived as distinctly different to previous therapy experiences, and participants noted that therapy could be intense and challenging, yet helpful later. Participants also reported that outside circumstances impacted on their progress in therapy and their voice‐hearing experience.

**Conclusions:**

The findings suggest that exposure‐based trauma‐focussed therapies may be beneficial for people who hear trauma‐related voices. However, this intervention can be intense and clinicians and consumers need to consider the timing of delivery, and pay attention to internal and external resources that can increase participants’ sense of safety.

**Practitioner points:**

- Imaginal exposure may be an effective intervention for people who hear voices that they perceive to be associated with a past traumatic event.
- Positive changes associated with the intervention may be highly variable between individuals, and encompass changes in sense of self, changes to internal states, and changes to voice‐hearing experience.
- Imaginal exposure interventions may involve some temporary discomfort and symptom exacerbation, which may affect the acceptability of the intervention. This needs to be considered in both future research and clinical delivery.

## Background

The experience of hearing voices in the absence of an external stimulus, also known as auditory hallucinations, often causes significant distress and disruption to a person’s life (McCarthy‐Jones, Marriott, Knowles, Rowse, & Thompson, 2013). Among current psychological treatments for hearing voices, Cognitive Behavioural Therapy for Psychosis (CBTp) is currently the best evidenced psychological intervention (National Institute for Clinical Excellence, 2014). However, CBTp has been critiqued for only producing small‐to‐moderate effect sizes (van der Gaag, Valmaggia, & Smit, 2014). Furthermore, psychological treatments developed to date have primarily targeted adaptation to hearing voices by reducing associated distress, rather than targeting the mechanisms presumed to underlie voice‐hearing (Thomas et al., 2014).

A key potential direction for the treatment of distressing voices is the use of trauma‐focussed interventions (Steel, 2015; Thomas et al., 2014). There is mounting evidence that trauma is a risk factor for developing psychotic symptoms, including voices (Brand, Rossell, Bendall, & Thomas, 2017) and theory and evidence point to the importance of posttraumatic stress symptoms and trauma memory processing in this relationship (Brand et al., 2020; Hardy, 2017; Steel, 2015). Trauma‐focussed interventions are evidence‐based psychological interventions that are effective in reducing posttraumatic stress symptoms (Bisson et al., 2007) and thus may be effective for treating voices that are driven by posttraumatic stress symptoms. Steel (2015) posits that auditory and visual hallucinations may be a form of re‐experiencing a traumatic event, but are not recognized as a memory due to rapid rather than integrated information processing during the event. As such, targeting trauma symptoms that are in some way connected to voice‐hearing experiences may assist in reducing voice‐hearing experiences or distress associated with the voices.

Sin and Spain (2017) conducted a meta‐analysis on psychological interventions for trauma for people with a diagnosis of psychosis, and found that psychological therapies were effective in reducing trauma‐related symptoms. Swan, Keen, Reynolds, and Onwumere (2017) presented a systematic review on the use of psychological treatments targeting post‐traumatic stress symptoms for people with a psychotic disorder. Consistent with Sin and Spain, the review found that trauma‐focussed psychological interventions were effective in reducing post‐traumatic symptoms in people with comorbid posttraumatic stress disorder (PTSD) and psychosis. A meta‐analysis by Brand, McEnery, Rossell, Bendall, and Thomas (2018) found that treatment of comorbid PTSD has a small but significant secondary effect on reducing positive symptoms of psychosis immediately after therapy; exposure‐based treatments (i.e. those that included direct exposure to the trauma memories and reminders) were particularly effective. Although specific effects on voices appeared limited, therapies targeting trauma memories specifically related to voices could have a more potent effect (Brand et al., 2018).

Paulik, Steel, and Arntz (2019) trialled the use of Imagery Rescripting for PTSD symptoms and voices, and focussed on traumatic memories related to voice‐hearing experiences. Imagery Rescripting involves the reimagining of traumatic events whereby the consumer imagines a new and safer ending to the memory (Paulik et al., 2019). The use of Imagery Rescripting was effective in improving PTSD symptoms and decreasing voice distress and frequency (Paulik et al., 2019). The study provides support for the use of trauma‐focussed interventions focussing on traumatic memories related to distressing voice‐hearing experiences.

Brand et al. (2021) conducted a case series of a six‐session imaginal exposure intervention for trauma‐related voices. The aim was to establish the feasibility and acceptability of delivering imaginal exposure as an intervention targeting relevant voice‐related trauma memories and to provide initial estimates of effects on voices. Established as an effective intervention component in the treatment of PTSD (e.g., Cusack et al., 2016), imaginal exposure involves repeated exposure to the trauma memory, in imagination, within the safety of the therapy room (Bourassa et al., 2020; Foa, Hembree, & Rothbaum, 2007). The imaginal exposure intervention was based on Foa's prolonged exposure manual (Foa et al., 2007), and was delivered in six sessions of 90 min each. In the first session, the therapist collaboratively explored links between traumatic events and content of voice‐hearing experiences. The rest of the intervention involved exposure to the trauma memory through repeated retelling of the traumatic event and listening to audio recordings of this between sessions. The traumatic events identified in the first session as having a link with the voices were the targeted memories for the rest of the intervention.

Quantitative findings from this case series showed high levels of satisfaction with the intervention; however, temporary discomfort and symptom exacerbation were common, and contributed to discontinuation in treatment for a minority (Brand et al., 2021). There was a large reduction in voice‐hearing severity at one‐month follow‐up, but individual response was highly variable (Brand et al., 2021; Brand, Hardy, Bendall, & Thomas, 2019). Brand et al. (2021) also reported large reductions in PTSD symptoms and the intrusiveness of the trauma memory (hypothesized mechanisms of action).

Understanding client experience of such interventions is critical to their implementation. Qualitative research is important in understanding client experience as it aids in amplifying participant voice, allowing participants to give feedback about the intervention in their own words (Corstens, Longden, McCarthy‐Jones, Waddingham, & Thomas, 2014). One reason for the underutilization of trauma‐focussed treatments for individuals with concurrent trauma and psychosis symptoms is practitioner concern around the safety of these therapies in individuals with psychosis. Practitioners may be particularly hesitant to undertake therapy involving exposure to trauma memories and reminders due to fears that this will worsen psychotic symptoms and increase risk (Becker, Zayfert, & Anderson, 2004; Gairns, Alvarez‐Jimenez, Hulbert, McGorry, & Bendall, 2015), even though this is not borne out by trial evidence. (e.g., Brand et al., 2018; van den Berg & Van der Gaag, 2012). Consequentially, people experiencing psychosis and trauma‐related symptoms may not receive optimal treatment (Keen, Hunter, & Peters, 2017). The use of qualitative methodology allows for an in‐depth understanding of experience that may not be possible with quantitative methodology (Povee & Roberts, 2014). Providing a deeper level of understanding of the experiences of individuals with psychosis participating in trauma‐focussed treatment may assist practitioners in feeling more confident in utilizing this approach for this population.

There is also a broader need to understand the client experience of psychological therapies. In psychological therapies one in 20 people report that therapy had a negative and lasting effect (Crawford et al., 2016). Examination of client experiences is particularly pertinent for exposure‐based treatments, given the potential tolerability difficulties, high drop‐out rate, and challenging nature of the intervention (Paulik et al., 2019).

Qualitative research is important in that it allows for detection of more subtle variations in benefits and adverse effects that may not be picked up by quantitative methodology (Horigian, Robbins, Dominguez, Ucha, & Rosa, 2010). The current study was a qualitative study nested within the Brand et al. (2021) case series of imaginal exposure for trauma‐related voices. It aimed to:

1. explore client experience of the imaginal exposure intervention;
2. identify beneficial and/or adverse effects of imaginal exposure for the treatment of distressing voices from participants’ perspectives;
3. understand overall tolerability of imaginal exposure.

## Method

### Study design

A qualitative study involving semi‐structured interviews was nested within the case series study. Participants had taken part in the intervention and were offered the opportunity to provide more information about their experience by participating in a semi‐structured interview. Ethics approval was granted by Swinburne University Human Research Ethics Committee. Themes of the interviews were analysed using thematic analysis, as per guidelines of Braun and Clarke (2006).

### Recruitment

Following treatment completion or termination, participants were invited to take part in an individual interview with a separate researcher (N.F.) to talk about their experience of the intervention. Participants who agreed to participate in the interview were contacted by N.F. who provided details about the study. The participants in the current study were those individuals who took part in the case series and who had verbally consented to participate in this interview.

Recruitment for the parent case series study (Brand et al., 2021) occurred by inviting people who were attending a specialist voices clinic (Thomas, Rossell, Farhall, Shawyer, & Castle, 2011) or who were on a hearing voices research participant registry. The study was also promoted in local clinical services and consumer groups. To be eligible for the case series study, participants needed to be adults experiencing current auditory hallucinations (as confirmed using item K6b of the MINI 7.02, Psychotic Disorders version; Sheehan et al., 1998), that were frequent and persistent (present for more than 6 months and occurring at least twice a week), have a history of traumatic events, and have made a conceptual link between past adverse experiences and their experience of voice‐hearing. All participants in the case series study were asked if they were willing to receive information about the qualitative study in their final session, including those who opted out before finishing the six sessions. The first author then contacted participants by telephone to provide details of the qualitative study.

### Interview schedule

An adapted version of the client change interview schedule (Elliott, Slatick, & Urman, 2001) was used as a semi‐structured guide during the interviews (Appendix S1). The purpose of the client change interview questionnaire is to obtain three types of information: changes experienced by clients over the course of therapy, clients' perceptions of the source of the changes, and difficult or hindering aspects of therapy (Elliott et al., 2001). The client change interview schedule was adapted in consultation with a panel consisting of community members with lived experiences of psychosis. The primary modification made was the addition of four questions: ‘What was it like going straight into the intervention?’; ‘How was your relationship with the therapist?’; ‘How did your experience of therapy compare with other therapy experiences?’; ‘Would you recommend this type of therapy to a friend in a similar situation?’. The interviewer also asked further clarifying questions to follow‐up individual experiences and allow for richer data led by participant perspectives.

### Procedure

Following the completion of the intervention, 10 participants took part in a single semi‐structured interview in which they spoke in detail about their experience of the intervention.

The interviews were conducted by a female postgraduate psychology student (N.F.) who was not involved in the implementation of the treatment and who had no prior relationship with participants. Participants were aware of the researcher’s discipline and student status, and that the qualitative research was being conducted as a component of the broader study. The interviews were conducted at the university campus where participants had received the treatment with no one else present. Following the completion of the interview participants were reimbursed with AUD $10 (US $8). The interviews ranged from 11 to 70 min (*M* 31 min, *SD* 18 min). All participants consented to having their interview audio recorded. The researcher recorded reflections including her observations and impressions after each, and was particularly mindful of potential biases in support of the use of psychological therapies given her disciplinary background. Transcripts were not returned to participants for comment/correction due to the anticipated burden, as participants were still involved in follow‐up assessments for the parent case series study.

### Qualitative analysis

The study utilized a realist framework, with an emphasis on understanding individuals’ experiences and perspectives, aided by an inductive approach where themes were identified from the data (Braun & Clarke, 2006). The interviews were analysed by the first author (N.F) within a Thematic Analysis framework as per Braun and Clarke’s (2006) six‐phase approach. Thematic analysis is a flexible method that has been widely used in health and wellbeing research (Braun & Clarke, 2014).

Phase One of the analysis involved “familiarising yourself with the data”, which was achieved by the first author manually transcribing each of the transcripts (Braun & Clarke, 2006). For Phase Two (‘generating initial codes’), initial codes were identified from the data using in vivo coding, which involves taking short phrases or words within the interview to create codes (Braun & Clarke, 2006; Saldana, 2013). In vivo coding was used with the aim of ensuring participants’ perspectives were accurately conveyed (Saldana, 2013). Next, for Phase Three (‘searching for themes’), codes were manually sorted into potential sub‐themes and themes using mind maps (Braun & Clarke, 2006). Sub‐themes were ultimately incorporated into larger themes. The coding process and decisions were reviewed with the co‐researchers (N.T., R.B., A.W.). An extract of the coding tree for one theme is shown in Table 1.

**Table 1:** Excerpt of coding tree for one theme

Codes	Subthemes	Theme
Thinking about past was a negative experience at the time. Process of imaginal exposure challenging at times Feeling distressed during therapy Therapy was intense	Subtheme: Therapy intense/stressful	Therapy an intense/stressful experience at the time, with benefits later Brief description: Participants commented that various aspects of the therapy were intense and stressful. For some participants, thinking about the past was what made the therapy stressful. For others, the repetition was stressful. Some participants commented that beginning therapy was particularly intense, and several commented that while therapy was difficult at the time, it was helpful in hindsight.
Hard to start therapy The more I talked about the event the easier it got Felt scared about going into treatment Imaginal exposure more manageable over time	Subtheme: Therapy initially very intense but became easier over time	
Helpful to talk about past even though it was difficult Imaginal exposure both beneficial and challenging simultaneously Confronting at time but glad I did it Therapy difficult but learnt a lot Therapy initially negative experience but turned out to be a positive thing	Subtheme: Repeating traumatic event difficult but helpful	

In Phase Four (‘reviewing themes’), the developing themes and their definitions were presented to a lived experience advisory panel. The discussion with the panel supported the proposed themes and resulted in refinements to theme descriptions. The interviewer presented preliminary themes to the panel with examples of codes taken from transcripts, and the panel suggested modifications to themes. For example, for theme three, the panel suggested that it needed to be more explicit that symptoms fluctuated in accordance with events outside of therapy, so the theme was altered to explicitly reflect this. Next, the first author collated all relevant data extracts within identified themes. The theme names and descriptions were revised and updated in Phase Five (‘defining and naming themes’), in consultation with co‐researchers for peer review and feedback. Consideration was given to issues including whether the sub‐themes and themes were well‐supported, and whether titles and descriptions were accurate, with an emphasis on capturing the essence of the theme (Braun & Clarke, 2006). These consultative processes helped to minimize the potential for the analysis to be overly influenced by the worldview of one analyst. While the number of participants was considered small, discussion in the research team resulted in consensus that no new themes appeared to be emerging from the interviews, suggesting that the analytical framework accommodated all of the relevant data. The write‐up of the analysis in Phase Six (‘producing the report’) was also reviewed by co‐researchers.

## Results

### Participant characteristics

Participant characteristics are shown in Table 2. Ten of the 15 participants from the parent study agreed to participate in the interview as part of the current study. All 10 interview participants had completed the imaginal exposure intervention within the three weeks prior to the interview. The five case series participants who did not choose to participate in an interview included three who withdrew from the intervention (two due to reported symptom exacerbation).

**Table 2:** Participant characteristics (*N* = 10)

	
Gender, *n* (%)	
Female	6 (60)
Male	4 (40)
Ethnicity, *n* (%)	
Caucasian	8 (80)
Hispanic	1 (10)
Other	1 (10)
Highest level of education, *n* (%)	
Primary	1 (10)
Secondary	2 (20)
TAFE/diploma	5 (50)
Undergraduate	2 (20)
Index traumatic event type, *n* (%)	
Childhood sexual abuse	2 (20)
Childhood emotional abuse	2 (20)
Childhood abuse (sexual, physical and emotional)	1 (10)
Adulthood sexual assault	2 (20)
Work‐related accident	1 (10)
Witnessing death of family member	1 (10)
Military trauma	1 (10)
Primary diagnosis, *n* (%)	
Schizophrenia	2 (20)
Schizoaffective disorder	2 (20)
Unspecified schizophrenia spectrum disorder	2 (20)
Bipolar I disorder	2 (20)
Mood disorder with psychotic features	1 (10)
Borderline personality disorder	1 (10)
Comorbid PTSD, *n* (%)	5 (50)
Comorbid borderline personality disorder, *n* (%)	3 (30)
Number of years had voices, *M* (SD)	16.25 (8.63)

### Results of thematic analysis

Four main themes were produced from the data: positive changes since therapy, changes in relationship to the outside world, therapy intense with benefits later, and a different approach with positives and negatives.

#### Theme One: Positive changes since therapy

All participants reported at least one positive change in themselves since the imaginal exposure therapy. These changes were often attributed to the intervention (*n* = 7). Some participants spoke of changes in their sense of self (*n* = 6). For example, Participant 4 reported, ‘It has been … helpful ’cause [I was] told not to blame myself for the traumatic event that happened.’ Participant 2 reported an increase in self‐compassion, and commented ‘being able to look at the content and stuff and think oh my God, you're doing really well, given what happened.’ Participant 7 reported feeling ‘more comfortable with myself’ following the therapy.

Some participants reported a reduction in internal negative feelings such as stress, anxiety, or depression, to varying degrees (*n* = 5). For example, Participant 2 reported feeling less stressed and depressed since therapy, and Participant 7 reported, ‘I don’t feel so intense now’. Participant 9 reported that they feel less hypervigilant, and Participant 10 reported ‘not having the urge to self‐harm as much’.

A smaller number of participants (*n* = 3) spoke about other positive changes to wellbeing, with a common theme of developing resilience or coping strategies in some aspect. For example, Participant 5 reported a sense of resilience in coping with emotions:

[Before the intervention] I was feeling, er, so [pause] really pushed down by the weight of my emotional baggage, and er, now, we've managed to defuse some of those, pump them full of helium [laughs] … so that they float at a reasonable distance and don’t just push me down by resting directly on me’.

Other positive changes related to coping or increased resilience included being able to ‘sit a little easier with the distressing stuff’ (Participant 8), and ‘[learning] techniques to manage the distress’ of the traumatic memory (Participant 6).

For some participants, improvements after the intervention came in the form of a reduction in their experience of hearing voices or distress associated with voices (*n* = 5). There was significant variability between participants in changes to their voice hearing experience after the intervention. Participants reported that the voices had become softer, weaker, or fainter, and one participant reported a total absence of voices following the intervention. Some participants reported increased tolerability of the voices. For example, Participant 3 commented: ‘ I'm more tolerant of the voices’ whilst not observing any changes in their frequency or content. Importantly, several participants reported no changes or very minimal changes in their voice hearing‐experience, such as Participant 4, who reported that the voices are ‘still the same as they have been’.

However, participants also noted fluctuations in voice‐hearing experience following the intervention (*n* = 2), as described by Participant 1, who noted that despite voices being ‘softer’ since the intervention in general, also said ‘yesterday, the voices were very strong: I couldn't sleep’ and that ‘they keep talking all the time, never stop, you know’.

Seven participants spoke of changes in the nature of their trauma memory since the intervention. More specifically, participants spoke about having greater clarity of the traumatic event since the intervention, and feeling like they have a better understanding of the events. Some participants spoke about how the traumatic memories felt more ‘in the past’ than they did before the intervention, with Participant 5 commenting, ‘… they do feel more their chronological age now … rather than I experiencing them as emotionally identical with what's currently happening.’

There was a sense that the intervention improved participants’ sense of cohesion of the traumatic memory, both in terms of the memory details and their understanding of the event. In some cases, this cohesion lessened the distress associated with the events, or made them more bearable. For example, Participant 6 reported:

I think that before I went through this it was just a big cluster of distressing kind of ideas that really didn't have much framework…um and so that sort of really helped me to sort of untangle that now and also found some techniques which have really helped in managing the distress.

When asked what they believed was the cause of the positive changes they had experienced since therapy, several participants identified that the intervention was the cause of one or more of the changes that they experienced (*n* = 7). While some participants were unsure of the aspects of the intervention that caused changes, some identified the repetition as causing the changes. Other participants attributed changes to the following factors: talking to the therapist; learning some techniques; change in feelings towards self; changes in response to others (e.g. reduction in social anxiety).

#### Theme Two: Therapy experience intense with benefits later

Participants often spoke about the intervention being intense or stressful, particularly at the beginning.

For some participants, thinking about the past made the intervention stressful. For example, Participant 1 commented: ‘the only negative thing I found is…because it reminds me of the past…I prefer not to think much about it’. For others, the repetition was difficult. Notably, Participant 6 reported experiencing a temporary exacerbation of voice‐hearing symptoms during therapy, which declined at the termination of therapy.

When I was doing the listening exercises I was experiencing a lot of, yeah, hearing voices and feeling distressed a lot…not a lot but more than when I started, and so now that I haven't been doing it for a few weeks it's declined again.

Some participants commented that beginning the intervention was particularly intense, but that it became easier over time (*n* = 4). Participant 7 reported, ‘The first three sessions were [in]tense but the fourth session was a bit [in]tense, but the fifth and the sixth session was easy.’ Around half of the participants stated that although the therapy was difficult and intense, it was ultimately helpful. For example, Participant 5 commented:

… it’s like going to a swimming hole and jumping off a ledge into the water… yes it’s scary, to begin with, and then it’s almost a feeling of elation afterwards

All participants who were interviewed had found the discomfort associated with the intervention to be tolerable enough to finish the six sessions of therapy.

#### Theme Three: Changes in relationship with the outside world

Participants spoke about how their functioning in the world was changed when they were undergoing the intervention (*n* = 3). For example, Participant 9 commented: ‘During it…I became more irritable’. Other participants described how they experienced increased difficulty in coping with relationships (Participant 2), or increased difficulty tolerating physical contact (Participant 6).

For other participants, their symptoms fluctuated in line with outside circumstances, both during and after the intervention (*n* = 3). For example, Participant 10 spoke about how initially after the intervention, their voice‐hearing experience had improved, but when outside circumstances became difficult, she experienced worsening of voice‐hearing symptoms. She reported: ‘For a little while afterwards, it was easier to rationalise what was real and what wasn't. It has been a bit harder over the last couple of weeks.’

#### Theme Four: A different approach with positives and negatives

All participants spoke about how the intervention was different to their previous therapy experiences, and some perceived other therapy experiences as avoiding discussion of these traumatic experiences (*n* = 3). Several alluded to the structured and focussed nature of the therapy (*n* = 5). For example, Participant 8 commented, ‘This is definitely more directed. This is more, ‘no messing about’, ‘let’s get right to the root of it’. The structured approach was appreciated by some participants. For example, Participant 8 commented, ‘I really liked, in a sense, that it was more direct, and there was a clear scope. ‘

Three participants expressed a desire for the incorporation of more supportive interactions in addition to the exposure intervention. For example, Participant 2 said that they felt it needed ‘a little bit more focus on checking in, even though the check‐in was definitely there, and structured, and a part of it, I think a little bit more focus on, and a little bit more time devoted to sort of unpacking check‐in, perhaps.’

Participants often talked about the intervention fitting into a broader context of ongoing treatment. Some participants were engaging with general mental health support during the treatment process (although not concurrent psychological therapy), while others talked about intending to engage in future support (*n* = 5). These participants tended to talk about feeling that their recovery journey was unfinished. Participant 5 commented: ‘I'm not entirely sorted…I'm still pretty out of sync with myself in a lot of ways.’

## Discussion

This study aimed to understand the experience of a trauma‐focussed imaginal exposure intervention for people who hear voices associated with a traumatic event. The qualitative design of the study provided an opportunity to understand details of peoples’ subjective voice‐hearing experience from participants’ own perspectives, providing a nuanced understanding of changes to voice‐hearing experiences after imaginal exposure therapy that goes beyond general satisfaction and perceived efficacy. Participants reported a variety of positive changes from the intervention, including a decrease in negative internal states, more positive sense of self, and new coping strategies to manage distress associated with traumatic memories. Participants also reported changes in their experiences of hearing voices, for example, experiencing voices as softer, or voices becoming more tolerable. Furthermore, participants commented that their memory of the traumatic event focussed on in therapy was more cohesive and integrated. Positive changes reported varied significantly between individuals, but all individuals reported at least one positive change since therapy. Participants consistently reported that the intervention was intense, particularly early on, but it was mostly perceived as tolerable and was perceived as helpful later on. Participants reported that their experience with imaginal exposure affected their capacity to engage with the outside world, and that vice versa, when adverse events were occurring in the outside world, this affected their progress in therapy, which has important implications for therapy delivery. Finally, the therapy was generally perceived as a contrast from other therapy experiences, with some participants favouring the structured and focussed approach, and others desiring more time devoted to supportive counselling as part of the therapy.

The general positive changes reported by participants are consistent with previous studies investigating outcomes of prolonged exposure therapy for people with PTSD. Alongside reductions in PTSD symptoms, studies have consistently reported decrease in depression symptoms, anxiety symptoms, and an increase in overall quality of life (van Minnen et al., 2015; Powers, Halpern, Ferenschack, Gillihan & Foa, 2010). Given that people experiencing psychosis are often excluded from studies exploring the efficacy of prolonged exposure for PTSD (van Minnen et al., 2015), the current results suggest that people with psychosis symptoms experience many of the same positive outcomes as individuals with PTSD without psychosis.

The finding that participants experienced benefits from the imaginal exposure is in line with the quantitative results of the parent study (Brand et al., 2021), which found that satisfaction with the therapy was high. The qualitative study design was helpful in identifying that consumers may experience benefits above and beyond changes in symptomology commonly measured in quantitative studies, such as changes to sense of self. The fact that some participants reported positive changes in their voice‐hearing experiences following the imaginal exposure intervention is also in keeping with the quantitative results of the case series (Brand et al., 2021), which indicated an overall reduction in voice severity following the intervention, but that individual response was variable. It appears that some people derive particular benefit from an exposure‐based trauma‐focussed therapy whereas others do not.

While the parent study (Brand et al., 2021) found an overall reduction in voice severity, the current study provided further understanding of the ways in which participants experienced this reduction. For some participants, the voices/noises became weaker or fainter. However for others, they experienced a greater tolerance of the voices, without experiencing a change in voice quality or decrease in frequency. This suggests that the minimization of voice‐hearing experience is variable between individuals, and that decreased distress associated with the voices may present in a number of different ways. It suggests that reducing the distress associated with hearing voices may not be limited to eliminating or even minimizing peoples’ voice‐hearing experience. The variability in experience between participants also suggests that response to trauma‐focussed imaginal exposure therapy is somewhat difficult to predict in regards to the effects on voice‐hearing experience. To date there has been some exploration of reasons for variation in response to trauma‐focussed therapies for voices, including the impact of dissociation during therapy (Paulik, Newman‐Taylor, Steel, & Arntz, 2020) and the degree of relationship between the content of the voice and the index traumatic event (Brand, Hardy, Bendall, & Thomas, 2019). Further exploration of the variability in response will help to inform targeted delivery of these interventions as well as effective measurement of relevant outcomes.

The finding that the imaginal exposure therapy was intense and stressful is consistent with the parent study ([masked] 2020), and is unsurprising, as the temporary emotional discomfort associated with exposure therapy across all contexts is noted in the literature (e.g. Olatunji, Deacon, & Abramowitz, 2009; Tong, Simpson, Alvarez‐Jimenez, & Bendall, 2017). However, the noted symptom exacerbation in some participants is in contrast to findings reported by van den Berg (2012), that prolonged exposure and eye movement desensitization and reprocessing therapy (EMDR) for people with comorbid PTSD and psychosis did not lead to exacerbation of psychotic or PTSD symptoms. The findings here suggest that some people may experience a short‐term exacerbation of psychosis symptoms while participating in exposure therapy that is specifically targeting trauma memories related to these psychotic symptoms.

While the emotional discomfort noted in the current study was unsurprising, the study has provided some additional insights into this experience of discomfort. Participants used words such as ‘intense’, ‘stressful’, ‘challenging’ and ‘confronting’ when describing some aspects of therapy. However, multiple participants also described how the therapy was ultimately helpful despite the distress or intensity, and one participant highlighted how there was a feeling of achievement once the therapy was completed. An underlying theme of therapy being difficult at the time, but ultimately helpful once therapy had finished was identified. Understanding the tolerability of the intervention was a key part of the study, and the insight that participants generally found it to be intense but manageable is an encouraging finding in light of the perceived challenges of trauma‐focussed exposure therapy (Olatunji et al., 2009; Tong et al., 2017).

The finding that memories became more coherent, detailed, and felt like they occurred more in the past suggests that for people with voices with some connection to a past traumatic event, prolonged imaginal exposure to the event memory may assist with the integration and elaboration of this memory. This is in line with existing theory around trauma memory processing in exposure therapy (Schnyder et al., 2015). According to Hardy (2017) and Steel (2015), traumatic memories may present as voices due to inadequate processing and contextualization at the time of the event, and voices may be a form of re‐experiencing a traumatic event. It follows that therapies targeting integration and contextualization of traumatic memories which are linked to voices may decrease or eliminate peoples’ experience of hearing voices related to that particular memory. This theory is partially supported by the current findings, as several participants noted that their voices felt softer. However this was not reported universally. This variability could reflect different mechanisms being involved for different people, and/or the particular memories being targeted in therapy not being sufficiently connected with their voice‐hearing experience.

The findings of the current study bear some similarities to the outcomes reported by Paulik et al. (2019), including a reduction in voice‐related distress. However, while Paulik et al. report changes to beliefs/appraisals about voices, this was not reported by participants in the current study. An explanation for this is that the Imaginal Exposure intervention did not explicitly focus on changing meanings attached to trauma memories, unlike Imagery Rescripting (as per Paulik et al., 2019).

### Clinical implications

The findings provide preliminary evidence for the therapeutic potential of trauma‐focussed treatments for trauma‐related voices, in so far as therapy may lead to positive changes in voice‐hearing experience, and people who receive this therapy may also experience other positive changes such as improvements in their sense of self, increased coping skills, and decreased anxiety/tension.

The intensity, and in some cases, symptom exacerbation reported by participants suggests that both clients and therapists need to be adequately prepared for trauma‐focussed imaginal exposure therapy. This includes ensuring clinicians are adequately trained to deliver this specialized form of therapy, and are able to manage the complexity of balancing exposure‐based therapy components with tolerability and broader therapeutic needs. It is recommended that informed consent for imaginal exposure targeting trauma‐related voices involves a discussion of the potential for symptom exacerbation and planning to manage this if it occurs. For example, consumers may benefit from accessing outside supports while undergoing the therapy. In light of the intensity of the therapy experience, the study findings highlight the need for consumers to have a supportive environment outside of therapy, including the absence of stressful home events that may impede progress and/or exacerbate symptoms.

Another clinical implication is the importance of considering the incorporation of emotion regulation components in the therapy in order to enable people to cope with the intervention. Paulik et al. (2020) discuss a range of strategies to prevent dissociation during Imagery Rescripting interventions, many of which were incorporated into the Imaginal Exposure intervention in the parent study to support coping (e.g. grounding techniques, reassurance of safety, and building trust with the therapist). The work of Paulik et al. highlights the importance of incorporating emotion regulation components into exposure‐based therapies for people who hear voices. Another tool to consider is stabilization, which involves ensuring individuals' safety, minimizing problems with self‐regulation, and improving psychological and emotional skills (Cloitre et al., 2012). Stabilization is currently utilized in some ‘phase‐based’ trauma interventions to support consumers to safely participate in exposure to traumatic memories (e.g. van Vliet, Huntjens, Dijk, & Jongh, 2018). There is ongoing debate about the necessity and benefit of stabilization (Cloitre et al., 2010, 2012; McFetridge et al., 2017; van Vliet et al., 2018). Clinicians should balance whether individuals might benefit from stabilization prior to undertaking imaginal exposure that targets any trauma‐related psychotic symptoms against whether this would unnecessarily delay a potentially potent component of therapy.

### Limitations

The intervention delivered in the case series was designed to provide a ‘proof of concept’ for the use of imaginal exposure as an intervention component for treating trauma‐related voices. As such, imaginal exposure was offered as a brief, standalone intervention. This may not be representative of the delivery of trauma‐focused therapy in practice in which it is more likely to be embedded within a broader therapy approach, and delivered over more sessions. Indeed, the full prolonged exposure protocol involves 8‐15 sessions, rather than the six sessions offered in the current study (Karlin et al., 2010). Thus, reflections from participants presented here relate specifically to undertaking imaginal exposure as a brief, discrete intervention. The experience of receiving imaginal exposure integrated within a broader therapy approach may be different.

Another limitation of the study was that the three people who terminated therapy prematurely as part of the case series chose not to take part in the semi‐structured interview. This precludes a detailed exploration of reasons for terminating the intervention. There is debate regarding the prevalence of dropout from exposure‐based versus other trauma‐focused therapies (Hembree et al., 2003) and people with PTSD and comorbid psychosis do not appear to have higher dropout rates from prolonged exposure than other PTSD populations (van den Berg et al., 2016; Hembree et al., 2003). The rate of dropout in the case series (Brand et al., 2021) was also not above those that would be expected in general PTSD populations. Nevertheless, the reasons for not completing prolonged exposure therapy are not well understood (Najavits, 2015) and this would have been a helpful addition to the literature.

The small sample size is a limitation of the study, and a larger sample size may have been useful in better understanding common versus uncommon responses to the intervention. Additionally, the study only included people who had been hearing voices for a long time. Consequentially, there is limited understanding of whether the intervention is received differently by people with recent onset of voices.

Some participants were receiving general mental health support during the intervention period (e.g. case management, medication), so it is possible some therapeutic benefits occurring during the period of intervention occurred due to supports separate to the intervention.

Finally, interviewees were conducted when participants had recently finished their therapy course, which limits the capacity for understanding the duration of the effects of therapy over time.

### Directions for future research

The current study suggests that the use of trauma‐focussed interventions such as imaginal exposure can produce positive changes for people who hear voices and have experienced a traumatic event. In light of the intensity of the intervention, future research could investigate whether tolerability of the exposure elements can be improved by including a stabilization phase, or integration within a broader trauma‐informed therapeutic approach. Future research could also explore other mechanistic targets in addition to trauma intrusions such as dissociation or hypervigilance (Hardy, 2017). Finally, in light of the variability of individual experience in the intervention, future research could focus on improving understanding about who is most likely to benefit from imaginal exposure therapy for hearing voices.

### Conclusion

The current study explored the experience and therapeutic effects of a trauma‐focussed intervention, imaginal exposure for hearing voices, from the perspective of people who received this intervention. Participants reported benefits from the intervention, including experiencing reduced distress or other positive changes in their voice‐hearing experience. Participants reported a variety of other positive changes, including improved sense of self, improved coping, and a decrease of negative internal states. Whilst experienced as beneficial, therapy was also experienced as intense, with some people identifying it as difficult at the time but helpful later. Future research could explore ways to increase the tolerability of the intervention, and there is also a need to develop better understanding of who is likely to benefit from imaginal exposure intervention. Overall, the findings indicate that imaginal exposure therapy is a promising intervention for consumers who hear voices related to a previous traumatic experience, although consideration of the intensity and timing of the delivery is needed.

## Conflicts of interest

All authors declare no conflict of interest.

## Author contributions

**Natalie Feary:** Conceptualization (equal); Data curation (equal); Formal analysis (equal); Investigation (equal); Methodology (equal); Project administration (equal); Writing – original draft (equal). **Rachel Brand:** Conceptualization (equal); Methodology (equal); Supervision (equal); Writing – review & editing (equal). **Anne Williams:** Methodology (equal); Supervision (equal); Writing – review & editing (equal). **Neil Thomas:** Conceptualization (equal); Methodology (equal); Supervision (equal); Writing – review & editing (equal).

## Supporting information


**Appendix S1**. Interview schedule

## Data Availability

The data that support the findings of this study are available from the corresponding author upon reasonable request.
