# Supplementary material for: ‘Like jumping off a ledge into the water’: A qualitative study of trauma‐focussed imaginal exposure for hearing voices
Source: Psychol Psychother. 2021 Nov 19;95(1):277–94. doi: 10.1111/papt.12372 (PMC9298761; doi:10.1111/papt.12372)
Supplement: Supplementary file 1 — Appendix S1. Interview schedule [file PAPT-95-277-s001.docx]

Supplementary Material

**“Like jumping off a ledge into the water”: A qualitative study of trauma-focussed imaginal exposure for hearing voices**

**Interview Schedule**

**1. General Questions**

What has therapy been like for you? How has it felt to be in therapy?

How are you doing now in general?

***Other treatment-specific questions***

What was it like going straight into treatment?

How was your relationship with the therapist?

How did your experience of this therapy compare with other therapy experiences?

Would you recommend this type of therapy to a friend in a similar situation?

**2. Changes**

What changes, if any, have you noticed in yourself since therapy started?

***Additional prompts if participant has not mentioned changes of interest:***

Have you noticed any changes in your experience of hearing voices since therapy started? If yes, can you describe these changes? *{interviewer to prompt re: voice content, voice frequency, distress re voice, relationship with voice, beliefs about voice}*

Have you noticed any changes in the memories that you have of the traumatic event that you focussed on in therapy? If yes, can you describe these changes? *{Interviewer to prompt re: frequency of intrusions, vividness of intrusions, nowness of intrusions, distress re intrusions, new cognitive insights re event]*

Additional prompt: *Have you noticed any physical changes?*

If there was a particular change, can prompt: *What was going on for you at the time of the change?*

**3. Attributions**

In general, what do you think has caused these various changes? In other words, what do you think might have brought them about? (Including things both outside of therapy and in therapy)

**4. Helpful Aspects**

Can you sum up what has been helpful about your therapy so far?

If needed: Please give examples (e.g., general aspects, specific events). What made these things helpful to you?

**5. Problematic Aspects**

What kinds of things about the therapy have been hindering, unhelpful, negative or disappointing for you? (For example, general aspects. specific events)

Were there things in the therapy which were difficult or painful but still OK or perhaps helpful? What were they?

Has anything been missing from your treatment? (What would make/have made your therapy more effective or helpful?)

**6.** **Suggestions**

Do you have any suggestions for us, regarding the research or the therapy?

Do you have anything else that you want to tell me?

Interview adapted from: Elliott, R., Slatick, E., & Urman, M. (2001). Qualitative change process research on psychotherapy: Alternative strategies. Psychologische Beitrage, *43*(3), 69-111.

**Consolidated criteria for reporting qualitative studies (COREQ) Checklist**

| **No. Item** | **Guide questions/description** | **Reported on Page #** |
| --- | --- | --- |
| **Domain 1: Research team and reﬂexivity** |  |  |
| *Personal Characteristics* |  |  |
| 1. Inter viewer/facilitator | Which author/s conducted the interview or focus group? | 7 |
| 2. Credentials | What were the researcher’s credentials? E.g. PhD, MD | 7 |
| 3. Occupation | What was their occupation at the time of the study? | 7 |
| 4. Gender | Was the researcher male or female? | 7 |
| 5. Experience and training | What experience or training did the researcher have? | 7 |
| *Relationship with participants* |  |  |
| 6. Relationship established | Was a relationship established prior to study commencement? | 7 |
| 7. Participant knowledge of the interviewer | What did the participants know about the researcher? e.g. personal goals, reasons for doing the research | 7 |
| 8. Interviewer characteristics | What characteristics were reported about the inter viewer/facilitator? e.g. Bias, assumptions, reasons and interests in the research topic | 7 |
| **Domain 2: study design** |  |  |
| *Theoretical framework* |  |  |
| 9. Methodological orientation and Theory | What methodological orientation was stated to underpin the study? e.g. grounded theory, discourse analysis, ethnography, phenomenology, content analysis | 8 |
| *Participant selection* |  |  |
| 10. Sampling | How were participants selected? e.g. purposive, convenience, consecutive, snowball | 6 |
| 11. Method of approach | How were participants approached? e.g. face-to-face, telephone, mail, email | 6 |
| 12. Sample size | How many participants were in the study? | 9 |
| 13. Non-participation | How many people refused to participate or dropped out? Reasons? | 9 |
| *Setting* |  |  |
| 14. Setting of data collection | Where was the data collected? e.g. home, clinic, workplace | 7 |
| 15. Presence of non-participants | Was anyone else present besides the participants and researchers? | 7 |
| 16. Description of sample | What are the important characteristics of the sample? e.g. demographic data, date | 9 |
| *Data collection* |  |  |
| 17. Interview guide | Were questions, prompts, guides provided by the authors? Was it pilot tested? | 6 |
| 18. Repeat interviews | Were repeat interviews carried out? If yes, how many? | 7 |
| 19. Audio/visual recording | Did the research use audio or visual recording to collect the data? | 7 |
| 20. Field notes | Were ﬁeld notes made during and/or after the inter view or focus group? | 7 |
| 21. Duration | What was the duration of the interviews or focus group? | 7 |
| 22. Data saturation | Was data saturation discussed? | 9 |
| 23. Transcripts returned | Were transcripts returned to participants for comment and/or correction? | 7 |
| **Domain 3: analysis and ﬁndings** |  |  |
| *Data analysis* |  |  |
| 24. Number of data coders | How many data coders coded the data? | 8 |
| 25. Description of the coding tree | Did authors provide a description of the coding tree? | 9 |
| 26. Derivation of themes | Were themes identiﬁed in advance or derived from the data? | 8 |
| 27. Software | What software, if applicable, was used to manage the data? | 8 |
| 28. Participant checking | Did participants provide feedback on the ﬁndings? | 8-9 |
| *Reporting* |  |  |
| 29. Quotations presented | Were participant quotations presented to illustrate the themes/ﬁndings? Was each quotation identiﬁed? e.g. participant number | 10, 11, 12, 13, 14, 15 |
| 30. Data and ﬁndings consistent | Was there consistency between the data presented and the ﬁndings? | 9 |
| 31. Clarity of major themes | Were major themes clearly presented in the ﬁndings? | 10-15 |
| 32. Clarity of minor themes | Is there a description of diverse cases or discussion of minor themes? | 10-15 |

Source: Tong, A., Sainsbury, P., Craig, J. (2007). Consolidated criteria for reporting qualitative research (COREQ): a 32-item checklist for interviews and focus groups. *International Journal for Quality in Health Care*, *19*(6), 349–357.
